# Pharmacologic Management of Central Fever in Patients With Acute Brain Injury

**DOI:** 10.31486/toj.25.0045

**Published:** 2025

**Authors:** Maria Turcanu, Ethan Levitch, Bobby D. Nossaman

**Affiliations:** ^1^ The University of Queensland Medical School, Ochsner Clinical School, New Orleans, LA; ^2^ Department of Anesthesiology and Perioperative Medicine, Ochsner Clinic Foundation, New Orleans, LA

**Keywords:** *Acetaminophen*, *antipyretics*, *baclofen*, *brain injuries*, *bromocriptine*, *dantrolene*, *diclofenac*, *drug therapy*, *fever*, *propranolol*, *temperature*

## Abstract

**Background:**

Central fever is a noninfectious increase in body temperature to >37.5 °C that commonly occurs in patients with acute brain injury. Because central fever has detrimental effects on the injured brain and is associated with prolonged neurointensive care unit stays, timely diagnosis and treatment are imperative. Management includes both pharmacologic and nonpharmacologic methods. However, the optimal pharmacologic approach for the management of central fever remains unclear, so we conducted a scoping review to evaluate the pharmaceutical therapies currently being used.

**Methods:**

We screened 183 articles from database searches of PubMed, Embase, and Cochrane. Information on study type, interventions, outcomes, and side effects were extracted and analyzed from 13 articles that met inclusion criteria.

**Results:**

Our literature search showed that acetaminophen, baclofen, baclofen with propranolol, bromocriptine, dantrolene, and diclofenac have been administered to patients with central fever. While most of the articles included in this review are case reports, a randomized clinical trial identified continuous diclofenac infusion as a promising intervention. Acetaminophen reduced fever in patients with central fever; however, when compared to placebo, no significant difference in core temperature reduction was observed. In the articles that included a discussion of side effects, hepatotoxicity was the most commonly mentioned adverse effect.

**Conclusion:**

Diclofenac emerged as the most evidence-supported therapy, backed by higher-quality studies and consistent central fever resolution with relatively few side effects. Because of the paucity of high-quality evidence, further research is needed to establish optimal treatment strategies with minimal adverse effects for patients experiencing central fever.

## INTRODUCTION

Central fever, a noninfectious increase in body temperature to >37.5 °C resulting from dysfunction of the central nervous system, commonly occurs in patients with traumatic brain injury (TBI), stroke, subarachnoid hemorrhage, or other neurologic insult.^1^ Central fever, typically identified in the neurointensive care unit, results from disruption of hypothalamic regulation of body temperature, leading to an unregulated thermic response.^1^ Distinguishing between central fever and fever caused by infection is essential to avoid inappropriate antibiotic use and delayed recognition of neurologic deterioration.^2^ In patients with intracerebral hemorrhage, central fever is linked to poor outcomes, especially in patients with larger hemorrhage volumes or intraventricular involvement.^3^

In patients with TBI, neurogenic fever can result from increased intracranial pressure caused by cerebral edema, potentially leading to further brain damage if not managed appropriately.^4^ Neurogenic fever is a type of central fever that is more closely associated with spinal cord injuries above T6.^5^ While the terms reflect distinct underlying mechanisms, they are often used interchangeably in clinical contexts and are considered to be synonymous for the purposes of this review.^1,5^

An autonomic storm, also known as paroxysmal sympathetic hyperactivity (PSH) or paroxysmal autonomic instability with dystonia (PAID), is a different clinical syndrome of episodic excessive sympathetic activity that involves tachycardia, hypertension, dystonia, and sometimes fever, most often seen in patients with severe brain injury and attributable to loss of cortical inhibition.^6^ Given their distinct features and management protocols, PSH and PAID are not explored in this review.

Initial management of central fever focuses on ruling out infectious causes, controlling temperature with antipyretics or external cooling methods when needed, and addressing the underlying neurologic condition to prevent further complications.^1,7^

The purpose of this review was to evaluate the pharmaceutical therapies used to manage central fever and to determine the most effective medications.

## METHODS

We conducted a literature search of the PubMed, Embase, and Cochrane databases on February 10, 2025. Search terms included relevant synonyms of keywords such as “pharmacologic therapy” and “central fever.” Complete search terms are listed in Table 1. Peer-reviewed human subject research articles and case reports published in the prior 20 years were eligible for inclusion. Animal studies and articles evaluating secondary causes of fever and nonpharmacologic interventions were excluded. Inclusion and exclusion criteria are listed in Table 2.

**Table 1.** Advanced Search Terms by Database

Database	Search Terms
PubMed	(Pharmacologic OR medication OR drug OR drugs OR “pharmacologic therapy” OR “drug therapy") AND ("central fever” OR “central pontine fever” OR “neurogenic fever” OR “brain injury-induced fever” OR “fever in brain injury” OR “traumatic brain injury AND fever") AND (Intervention OR management OR outcomes OR treatment OR therapy OR “clinical outcomes") NOT (“pediatrics” OR “neonates” OR “children” OR “child” OR “kids") NOT (“sepsis” OR “infection” OR “viral infection” OR “virus” OR “infectious fever") NOT (“animals"[MeSH] NOT “humans"[MeSH]) AND ((adaptiveclinicaltrial[Filter] OR casereports[Filter] OR clinicaltrial[Filter] OR controlledclinicaltrial[Filter] OR guideline[Filter] OR introductoryjournalarticle[Filter] OR meta-analysis[Filter] OR multicenterstudy[Filter] OR observationalstudy[Filter] OR randomizedcontrolledtrial[Filter] OR review[Filter] OR systematicreview[Filter] OR technicalreport[Filter]) AND (fft[Filter]) AND (2015:2025[pdat])) AND ((adaptiveclinicaltrial[Filter] OR casereports[Filter] OR clinicaltrial[Filter] OR controlledclinicaltrial[Filter] OR guideline[Filter] OR introductoryjournalarticle[Filter] OR meta-analysis[Filter] OR multicenterstudy[Filter] OR observationalstudy[Filter] OR randomizedcontrolledtrial[Filter] OR review[Filter] OR systematicreview[Filter] OR technicalreport[Filter]) AND (fft[Filter]) AND (2015:2025[pdat]))
Embase	(pharmacologic OR medication OR drug OR drugs OR 'pharmacologic therapy' OR 'drug therapy') AND ('central fever' OR 'neurogenic fever' OR 'brain injury-induced fever' OR 'fever in brain injury' OR 'traumatic brain injury and fever') AND (intervention OR management OR outcomes OR treatment OR therapy OR 'clinical outcomes') NOT (pediatrics OR neonates OR children OR child OR kids) NOT (sepsis OR infection OR 'viral infection' OR virus OR 'infectious fever')
Cochrane	(Pharmacologic OR Medication OR Drug OR Pharmacotherapy) AND (“Central fever” OR “Central pontine fever” OR “Neurogenic fever” OR “Brain injury” OR “Traumatic brain injury”) AND (Intervention OR Management OR Treatment OR Therapy OR Outcome OR Prognosis OR Mortality OR Morbidity) NOT (Sepsis OR Infection OR Bacterial OR Viral OR Virus OR Fungal OR “Systemic inflammatory response syndrome” OR “Pediatrics” OR “Children” OR “Neonates” OR “Infants” OR “Newborn” OR “Animal model” OR “Rodent” OR “Mouse” OR “Mice” OR “Rat” OR “Rats” OR “Canine” OR “Dog” OR “Primate” OR “Monkey”)

**Table 2.** Inclusion and Exclusion Criteria for Screened Studies

Criterion	Explanation	Rationale for Inclusion or Exclusion in the Review
Inclusion		
Last 20 years	Articles were published within the last 20 years.	Focus is on the most contemporary and relevant evidence.
Pharmacologic intervention	Studies evaluated pharmacologic treatment of central fever.	Focus is on managing central fever rather than preventing it.
Adult population	Patients were aged 18 years and older.	Focus is on adult patients rather than the pediatric population.
Exclusion		
Nonpharmacologic	Studies evaluated nondrug interventions (eg, cooling, physical therapy).	Focus is on pharmacologic treatments only.
Secondary causes	Studies evaluated fever attributable to infections, inflammatory conditions, or other identifiable causes.	Focus is on central fever rather than fever caused by other conditions.
Animal studies	Studies were conducted on animal models rather than humans.	Focus is on clinical applications in humans.
Paroxysmal sympathetic hyperactivity or paroxysmal autonomic instability with dystonia	These clinical syndromes have overlapping causes with central fever.	Focus is on the treatment of central fever only.

Articles were imported to Covidence (Veritas Health Innovation Ltd) that removed duplicates and facilitated screening by 2 reviewers (EL and MT) from February 10, 2025, to February 25, 2025. Following title and abstract screening, disagreements about inclusion were resolved via group discussion with a senior critical care physician (BDN).

## RESULTS

The advanced keyword search identified 183 possible articles, and screening reduced the list to 13 eligible articles. Data extracted from the 13 articles are summarized in Table 3.^8-20^

**Table 3.** Summary of Included Studies

Study	Intervention	Study Type	Dosage, Modality	Central Fever Resolution	Outcome	Side Effects Discussed
Saxena et al, 2015^9^	Acetaminophen	Randomized clinical trial	1 g IV in mannitol 3.85 g solution every 4 hours	Yes	No significant difference from placebo	Monitored for hepatotoxicity
Chorostecki et al, 2023^8^	Acetaminophen	Case series	650 mg oral acetaminophen at variable intervals; 300 mg/15 mg acetaminophen with codeine	Yes	Monotherapy less effective than dual therapy	Hepatotoxicity, renal toxicity
Lee et al, 2014^11^	Baclofen	Case report	30 mg/day oral, increased to 60 mg/day	Yes	Successful treatment	Not reported
Sengupta et al, 2019^10^	Baclofen	Case report	20 mg/day	Yes	Successful treatment	Not reported
Poudel et al, 2023^12^	Baclofen with propranolol	Case report	10 mg every 8 hours orally (baclofen), 20 mg every 12 hours orally (propranolol); baclofen increased to 20 mg and both given every 8 hours	Yes	Successful treatment	Heart rate decrease (bradycardia not specified)
Yu et al, 2013^13^	Bromocriptine	Case report	2.5 mg/day for 3 days, increased to 5 mg/day	Yes	Successful treatment	Not reported
Reeder et al, 2018^16^	Bromocriptine	Retrospective cohort	Not reported	Yes	No significant difference in time to fever cessation in patients who received bromocriptine vs those who did not	Not reported
Perez et al, 2023^15^	Bromocriptine	Retrospective cohort	Median 7.5 mg/day (range, 2.5 to 40 mg)	Yes	Successful treatment	Not reported
Amin et al, 2024^14^	Bromocriptine	Retrospective cohort	Median 8 mg/day	Yes	Successful treatment	Hepatotoxicity, drug-induced liver failure, bradycardia
Kuboyama et al, 2024^17^	Dantrolene	Case report	40 mg IV	Yes	Successful treatment	Not reported
Cormio and Citerio, 2007^18^	Diclofenac	Randomized clinical trial	0.2 mg/kg IV loading dose (30 minutes), then continuous infusion of 75 mg in 50 mL saline, titrated based on temperature response (range, 0.004 to 0.08 mg/kg/h)	Yes	Significantly shorter fever resolution time compared to control	Impaired hepatic and renal function, hemorrhagic events^a^
Schiefecker et al, 2013^20^	Diclofenac	Observational	75 mg IV in 100 mL saline	Yes	Maximum reduction of temperature achieved in 5.5 hours	Cerebral perfusion pressure decrease, hypotension, hypoxia
Picetti et al, 2016^19^	Diclofenac	Prospective cohort	12.5 mg intramuscular single dose	Yes	Significant reduction in body temperature in 2 hours	Cerebral perfusion pressure decrease, hypotension

^a^The listed side effects were not observed in patients enrolled in the study but were discussed as being commonly associated with the use of nonsteroidal anti-inflammatory drugs.

IV, intravenous.

The selected studies examined the use of acetaminophen, an analgesic and antipyretic^8,9^; baclofen, a GABA-B agonist, with and without propranolol, a nonselective beta receptor antagonist^10-12^; bromocriptine, a dopamine agonist^13-16^; dantrolene, a skeletal muscle relaxant that inhibits calcium release from the sarcoplasmic reticulum^17^; and diclofenac, a nonsteroidal anti-inflammatory drug (NSAID).^18-20^ Although the patients in the studies had variable primary brain injuries and fever reduction times, each intervention achieved resolution of central fever (oral temperature <37.5 °C).^1^

### Acetaminophen

Acetaminophen used for the management of neurogenic fever demonstrated variability in dosage, administration timing, modality, and outcomes across the 2 identified studies.^8,9^ In a case series, Chorostecki et al reported 4 patients who received 650 mg oral acetaminophen and 1 patient who received 650 mg oral acetaminophen alternated with 300 mg/15 mg acetaminophen/codeine and concluded that monotherapy acetaminophen was less effective compared to the alternating dual therapy.^8^ In a randomized clinical trial, Saxena et al did not find a statistically significant temperature reduction (*P*=0.09) in patients who received 1 g intravenous (IV) acetaminophen vs patients who received placebo (0.9% sodium chloride) administered every 4 hours during a 72-hour period.^9^

### Baclofen

Three case reports documented the successful use of baclofen in the management of central fever.^10-12^ Dosages varied between 10 mg and 60 mg per day. Sengupta et al^10^ reported resolution of central fever within 2 days of administration, while Lee et al^11^ reported resolution in 3 days. Poudel et al reported fever resolution after 10 days of treatment and noted that combining propranolol with baclofen provided heart rate regulation and enhanced antipyretic efficacy compared to baclofen alone.^12^

### Bromocriptine

In the bromocriptine studies, daily dosages varied widely: from 2.5 mg to 40 mg.^13-16^ A case report by Yu et al reported successful resolution of central fever following a week of treatment with bromocriptine.^13^ A retrospective cohort study by Amin et al noted significant reduction in body temperature (*P*=0.005) measured before and 2 hours after administration of bromocriptine in 30 patients.^14^ Perez et al found similar results, with significant reductions in body temperature measured before and after bromocriptine administration in 33 patients at 24 hours (*P*=0.039), 48 hours (*P*=0.004), and 72 hours (*P*<0.001).^15^ Reeder et al, however, found no significant difference in time to fever cessation in patients who received bromocriptine vs patients who did not receive bromocriptine (*P*=0.61) in a retrospective cohort study of 26 patients with subarachnoid hemorrhage.^16^

### Dantrolene

Kuboyama et al documented successful resolution of fever in a patient with TBI 1 day after administration of dantrolene. A single 40-mg dose reduced body temperature and resolved shivering that was resistant to propofol, midazolam, and fentanyl.^17^

### Diclofenac

Three studies examined the efficacy of diclofenac in central fever management.^18-20^ In a randomized clinical trial of 22 patients, Cormio and Citerio compared continuous low-dose IV diclofenac sodium administration to IV boluses of NSAIDs (diclofenac, ketoprofen, or paracetamol according to physician preference) and found that the diclofenac sodium group had significantly fewer minutes of fever in each day of the 6-day treatment period (*P*=0.0005 to *P*=0.02) compared to the control group.^18^ Picetti et al found a significant reduction (*P*<0.001) in core body temperature measured with a bladder probe before and 2 hours after administration of a single 12.5-mg intramuscular dose of diclofenac to 30 patients with acute brain injury.^19^ In an observational study of 21 patients, Schiefecker et al founded that IV diclofenac effectively reduced body temperature in 91% of interventions, with a significant body temperature reduction (*P*<0.001) 5.5 hours after administration.^20^

## DISCUSSION

Elevated body temperature in brain-injured patients disrupts the blood-brain barrier, increasing excitatory amino acid release, elevating free radical production, and consequently exacerbating neuronal injury.^1^ Untreated central fever in hospitalized patients has been associated with extended length of stay and increased mortality.^2^ These outcomes underscore the importance of promptly identifying and managing central fever to mitigate potential harm.^2^

Acetaminophen, commonly used for general fever reduction, exerts an antipyretic effect by inhibiting prostaglandin synthesis centrally via cyclooxygenase (COX) inhibition, particularly COX-2 in the hypothalamus.^21^ However, in the context of central fever, which commonly involves hypothalamic injury and disrupted thermoregulation, the effectiveness of acetaminophen is often limited because of its dependence on an intact hypothalamic response.^8^ The Saxena et al randomized clinical trial found no significant difference between acetaminophen and placebo in fever reduction in neurologically injured patients, suggesting limited efficacy in central fever.^9^ Chorostecki et al found that dual therapy with acetaminophen and codeine was more efficacious than acetaminophen alone, but only a single patient in their case series was treated with the dual therapy.^8^

Baclofen, a GABA-B receptor agonist, has been proposed to treat central fever by modulating central neurotransmitter release. The rationale lies in the ability of baclofen to suppress excessive excitatory activity in the hypothalamus, which may play a role in thermoregulatory dysfunction.^11,12^ The combination of baclofen and propranolol demonstrated an additive benefit of heart rate regulation compared to baclofen alone in a patient with central fever. The synergistic effect of the combined medications, which mediated tachycardia and enhanced temperature reduction, merits further investigation to strengthen the evidence base for the specified therapeutic approach. The evidence we found in our literature search that supported baclofen as a treatment for central fever was limited to case reports, showing weak generalizability and preventing robust conclusions about the efficacy and safety profile of the drug in this setting.^10-12^

Bromocriptine, a dopamine agonist, may restore dopaminergic balance within the hypothalamus, potentially correcting the thermoregulatory dysregulation seen in central fever.^13^ The use of bromocriptine in the management of central fever showed promise in 2 retrospective studies^14,15^ and 1 case report.^13^ Although a third retrospective study found that central fever resolved after bromocriptine administration, the study did not find a significant difference in the time to fever cessation among the patients who received bromocriptine vs the patients who did not.^16^ These inconsistent findings were paired with a lack of side effect profile reporting.^16^ The retrospective study by Amin et al was the only article that acknowledged adverse impacts with bromocriptine, noting hepatotoxicity and bradycardia.^14^ The risk-benefit profile of bromocriptine in the setting of central fever is unclear, given that 3 of 4 articles did not report side effect information.^13,15,16^ Evaluation through prospective clinical trials is therefore warranted to clarify the role of bromocriptine in central fever management.

Dantrolene is a skeletal muscle relaxant primarily used in malignant hyperthermia and neuroleptic malignant syndrome.^22^ Dantrolene use to treat central fever is theoretically appealing when hyperthermia is driven by excessive muscle activity or rigidity, but dantrolene does not directly address hypothalamic dysfunction.^22^ The evidence for dantrolene efficacy in treating central fever is primarily extrapolated from related hyperthermic syndromes, limiting clinical relevance.^22^ However, Kuboyama et al found that a continuous infusion of dantrolene was successful in treating fever in a patient with a subdural hematoma.^17^ The effectiveness of dantrolene to also resolve refractory shivering in the patient provided a 2-fold benefit, suggesting its potential as an adjunct therapy for patients with central fever and concomitant shivering.^17^

The NSAID diclofenac showed the most consistent results in the literature we reviewed. In 3 studies, various routes and doses were associated with significant reductions in body temperature.^18-20^ Two studies evaluating intramuscular and IV bolus doses of diclofenac identified the same side effects of reduced central cerebral pressure and hypotension, stressing the importance of close monitoring.^19,20^ Notably, Cormio and Citerio found that continuous infusion of IV diclofenac significantly shortened fever resolution time compared to bolus dosing of diclofenac or other NSAIDs.^18^ Although no documented side effects were observed among test subjects in their randomized clinical trial, Cormio and Citerio discussed known and common side effects of the drug, as noted in Table 3.^18^ Furthermore, continuous diclofenac infusion demonstrated superior hemodynamic stability vs bolus administration, providing support for continuous infusion that allows for steadier dosing and closer titration monitoring.^18^

Based on study design and methodology, the diclofenac studies demonstrated the highest level of evidence of the 5 pharmacologic interventions reviewed.^18-20^ Diclofenac use was supported by a randomized clinical trial, a prospective cohort study, and an observational study, all of which provided clinically significant evidence.^18-20^ In contrast, Saxena et al^9^ showed no significant benefit with acetaminophen vs placebo in a randomized clinical trial, and all other medication studies were lower-level evidence formats^8,10-17^ based on the hierarchy of evidence quality.^23^ Our findings indicate that diclofenac currently is the most evidence-supported option for managing central fever in neurocritical care, although its use requires careful dose titration and monitoring because of potential hemodynamic side effects.^18-20^

This review highlights the lack of standardized pharmacologic approaches to central fever and the reliance on small-scale data. While diclofenac stands as the preferred evidence-supported intervention,^18-20^ the limited data for other agents with plausible central mechanisms of action and desirable outcomes signal a need for targeted clinical trials.

### Limitations

Limitations to this study include the low number of quality studies available, which restricts generalizability. Additionally, the different dosages used across studies of the same drug further complicate the ability to draw definitive conclusions regarding optimal pharmaceutical intervention. Furthermore, some studies did not report side effects, introducing potential reporting bias. The lack of data negatively impacts the interpretation of safety outcomes and limits comprehensive analysis. To optimize treatment strategies and improve patient outcomes, high-quality standardized research is essential.

## CONCLUSION

The management of central fever is a clinical challenge because of its resemblance to infectious fever and potential impact on neurologic outcomes. Effective management hinges on accurate differentiation of central fever from infectious fever and the administration of appropriate medications in patients with acute brain injury. This scoping review underscores the variability in pharmacologic approaches, highlighting the need for standardized treatment guidelines based on robust evidence. Among the available options, diclofenac was identified as the intervention with the strongest supporting evidence based on consistent findings in studies with a higher level of evidence compared to the studies of other agents. However, to optimize treatment strategies and improve patient outcomes, high-quality, standardized research is essential.

## Notes

*This article meets the Accreditation Council for Graduate Medical Education and the American Board of Medical Specialties Maintenance of Certification competencies for Patient Care and Medical Knowledge.*
